# Model of Tumor Dormancy/Recurrence after Short-Term Chemotherapy

**DOI:** 10.1371/journal.pone.0098021

**Published:** 2014-05-20

**Authors:** Shenduo Li, Margaret Kennedy, Sturgis Payne, Kelly Kennedy, Victoria L. Seewaldt, Salvatore V. Pizzo, Robin E. Bachelder

**Affiliations:** 1 Department of Pathology, Duke University Medical Center, Durham, North Carolina, United States of America; 2 Department of Medicine, Duke University Medical Center, Durham, North Carolina, United States of America; Florida International University, United States of America

## Abstract

Although many tumors regress in response to neoadjuvant chemotherapy, residual tumor cells are detected in most cancer patients post-treatment. These residual tumor cells are thought to remain dormant for years before resuming growth, resulting in tumor recurrence. Considering that recurrent tumors are most often responsible for patient mortality, there exists an urgent need to study signaling pathways that drive tumor dormancy/recurrence. We have developed an *in vitro* model of tumor dormancy/recurrence. Short-term exposure of tumor cells (breast or prostate) to chemotherapy at clinically relevant doses enriches for a dormant tumor cell population. Several days after removing chemotherapy, dormant tumor cells regain proliferative ability and establish colonies, resembling tumor recurrence. Tumor cells from “recurrent” colonies exhibit increased chemotherapy resistance, similar to the therapy resistance of recurrent tumors in cancer patients. Previous studies using long-term chemotherapy selection models identified acquired mutations that drive tumor resistance. In contrast, our short term chemotherapy exposure model enriches for a slow-cycling, dormant, chemo-resistant tumor cell sub-population that can resume growth after drug removal. Studying unique signaling pathways in dormant tumor cells enriched by short-term chemotherapy treatment is expected to identify novel therapeutic targets for preventing tumor recurrence.

## Introduction

Despite the apparent efficacy of chemotherapy in “shrinking” primary tumors, chemotherapy-resistant tumor cells are thought to contribute to future tumor recurrence, the leading cause of patient mortality [1]. The identification of proteins that confer chemotherapy resistance has historically relied on studies of signaling pathways supported by tumor cells subjected to long-term, high dose drug selection [2], [3]. These long-term selection models select for mutations/epigenetic modifications that result in acquired expression/activity of proteins involved in therapy resistance. The clinical relevance of these long term selection models remains controversial [4].

Other models propose that tumors are heterogeneous, consisting of therapy-sensitive and therapy-resistant tumor cell subpopulations [5], [6], [7], [8], [9], [10]. According to these models, following chemotherapy treatment, chemo-resistant tumor cells exist in a dormant (sleeping) state for many years before resuming growth, resulting in tumor recurrence. Methods are needed to enrich for dormant tumor cells, allowing for studies of their unique signaling properties. Such studies will be critical to defining logical therapeutic targets for preventing tumor recurrence.

Using short term chemotherapy treatment to enrich for drug-resistant tumor cells, we have developed an *in vitro* model of tumor recurrence. In this model, short-term exposure of breast and prostate tumor cells to clinically-relevant chemotherapy classes/doses enriches for a population of slow-cycling (dormant) tumor cells. Chemotherapy-enriched dormant tumor cells resume proliferation approximately ten days after chemotherapy withdrawal, forming colonies resembling a tumor recurrence. Colonies emanating from chemotherapy-enriched dormant cells exhibit increased resistance to the original chemotherapy insult, similar to recurrent tumors in cancer patients. Contrasting with evolution models of therapy resistance, the existence of drug-resistant tumor cell subpopulations in the original tumor suggests that we can effectively eliminate tumor recurrence by implementing combination therapies [chemotherapy (targeting proliferative cells)+therapy targeting drug-resistant tumor cells].

## Materials and Methods

### Cell Culture/Reagents

SUM159 cells were obtained from Duke Cell Culture Facility and maintained in Ham’s F-12 medium containing 5% heat-inactivated FBS, 5 µg/ml insulin, and 1 µg/ml hydrocortisone. DU145 prostate cancer cells were obtained from the Duke Cell Culture Facility and maintained in RPMI 1640 containing 10% heat-inactivated FBS.

### Time Course- Cell Death Following Acute Chemotherapy Treatment

SUM159 were incubated with doxorubicin (1 µM) for 2 d, after which chemotherapy was removed, and new media added. Photographs were taken using an Olympus inverted microscope with a Canon EOS Rebel T4I. Final magnifications were 4X and 10X. Viable cell number was determined by performing trypan blue stains on cells harvested at 6 h, d1, d2, d3, and d7 post-chemotherapy treatment. Alternatively, DU145 tumor cells were incubated with docetaxel (10 nM). Chemotherapy was removed after 4 d. Viable cell number was determined as above for chemotherapy-treated SUM159 cells.

### Time Course- Regrowth of Chemo-residual Tumor Cells

Six days after chemotherapy removal, SUM159 cells were harvested with trypsin-EDTA, and replated in 96 well plates (1000 cells/well). Tumor cell proliferation was assessed on a daily basis by measuring thymidine uptake. For the DU145 model, DU145 cells were harvested with accutase six days after chemotherapy removal, and replated in 96 well plates (1000 cells/well). Tumor cell proliferation was assessed on a daily basis by measuring thymidine uptake.

### Evolution of “Recurrent” Colonies

SUM159 dormant cells were harvested 5–6 d after chemotherapy removal with trypsin-EDTA, and re-plated in 6-well plates (10^5^ cells/well). Media was changed every 3–4 d. Recurrent colonies (d18–d22) were stained with crystal violet and colonies containing >50 cells were counted. DU145 dormant cells were harvested with accutase 6 d after chemotherapy removal and re-plated in 6-well plates (2.5×10^3^ cells/well). Media was changed every 5–6 d. Recurrent colonies were stained with crystal violet on d22 and counted using the GelCount.

### Western Blots

Cells were harvested using trypsin-EDTA, washed with PBS, incubated in RIPA buffer on ice for 20 min, and then subjected to high speed centrifugation to obtain total cellular protein in the soluble fraction. For nuclear protein extraction, harvested cells were first incubated in cytosolic lysis buffer (10 mM HEPES, 10 mM KCl, 1.5 mM MgCl_2_, 0.5% NP40, and proteinase inhibitors) on ice for 20 min, centrifuged, and the supernatants were collected as cytosolic protein lysates. The residual pellets were washed with cytosolic lysis buffer once, and then incubated in nuclear lysis buffer (50 mM TRIS, 1% SDS, and proteinase inhibitors) plus Benzonase (Sigma, St. Louis, MO) on ice for 20 min. The supernatants after centrifugation were collected as nuclear protein extracts. Protein concentrations were determined by BCA assay. Equivalent amounts of protein were subjected to SDS-polyacrylamide gel electrophoresis (PAGE) and immunoblotted with the following primary antibodies, followed by the approprimate species IRDye-conjugated secondary antibody (Invitrogen): p21 (Cell Signaling), GAPDH (GenScript), Actin (Sigma). Proteins were detected using Odyssey infrared imaging system (LI-COR, Lincoln, NE).

### Thymidine Uptake

Cells were plated in 96-well plates (2×10^3^ cells/well). After overnight incubation, cells were incubated with 0.5 µCi/well [Methyl-^3^H]-Thymidine (Perkin Elmer) for 4–6 hs before harvesting onto glass-fiber filters. [^3^H]-Thymidine incorporation was measured as counts per minute (CPM) using a Tri-Carb 2100TR time-resolved liquid scintillation counter (Perkin Elmer).

### Alamar Blue

Cells were plated in 96-well black, clear bottom plates (2×10^3^ cells/well) in 100 µl complete medium. After 6 h, 10 µl/well alamarBlue (Life Technologies) reagent was added and, after 3 hs, fluorescence was measured using a Cytation3 plate reader (BioTek).

### PKH Labeling Study

SUM159 and DU145 cells were labeled using the PKH26 Red Fluorescent Cell Linker Kit (Sigma) according to the manufacturer’s instructions. The labeled SUM159 cells were treated with doxorubicin (1 µg/ml) to generate chemotherapy enriched dormant cells, as described above. Likewise, PKH26-labelled DU145 were treated with docetaxel (10 nM) to generate chemotherapy-enriched dormant cells, as described above. Labelled cells were detected using the Guava EasyCyte Plus flow cytometer (Millipore).

### Measuring Chemotherapy Sensitivity of Recurrent Tumor Cells

SUM159 and DU145 “recurrent” colonies (as described above) were re-plated in T75 tissue culture flasks and grown as a monolayer. Parental tumor cells and recurrent tumor cells were plated in 96-well plates (2×10^3^ cells/well). After overnight incubation, cells were incubated with media only, doxorubicin, or docetaxel at the indicated concentrations for 2 d. [Methyl-^3^H]-Thymidine was added (0.5 µCi/well) 6 h before harvesting onto glass-fiber filters. [^3^H]-Thymidine incorporation was measured as described above. Data were reported as fold change relative to cells cultured in media alone.

## Results

**Figure 1:**
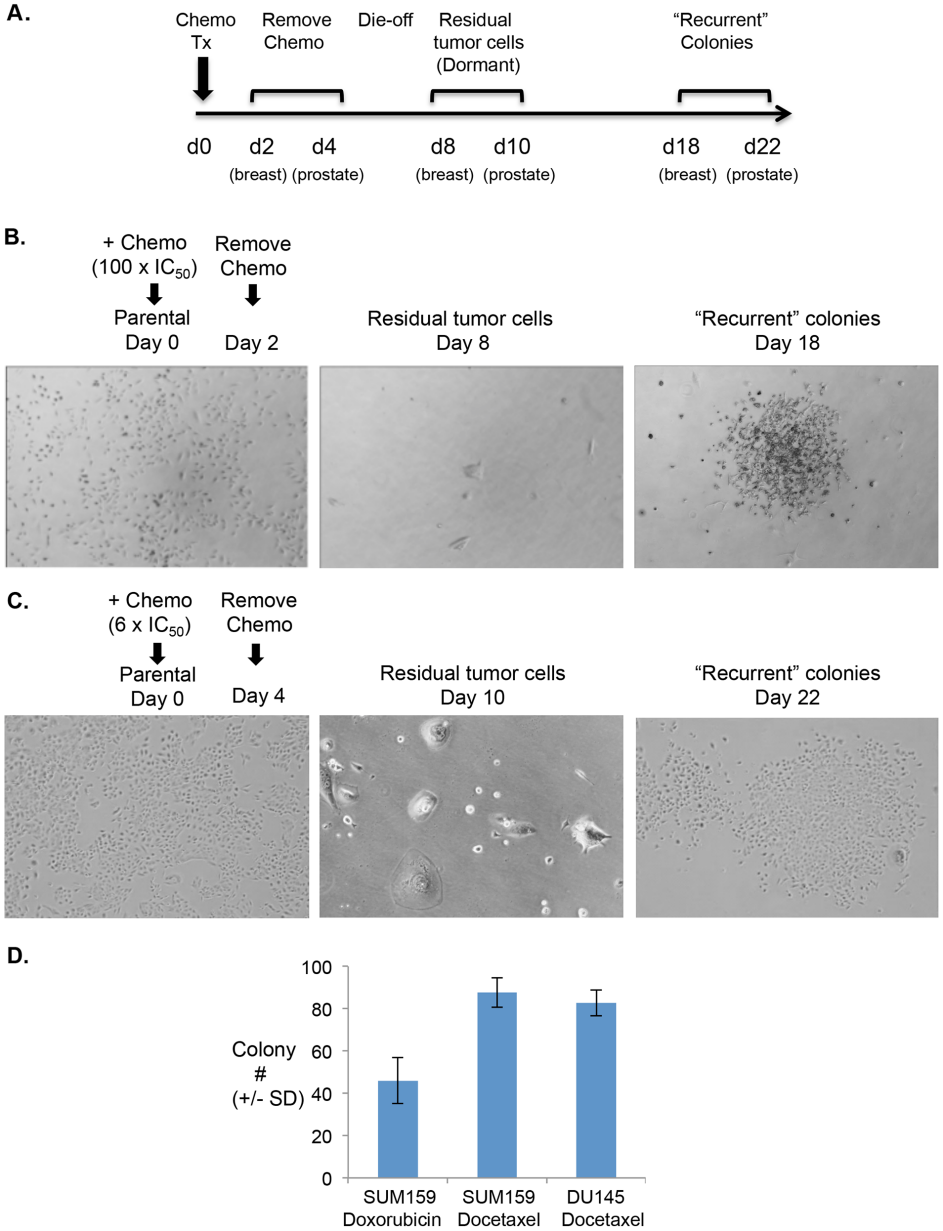
*In vitro* model of tumor dormancy/recurrence after short-term chemotherapy treatment. **A.** Schematic of experimental tumor dormancy/recurrence model. Breast (SUM159) or prostate (DU145) tumor cells were treated short term (breast 2 d; prostate 4 d) with chemotherapy *in vitro*. After 8 d (breast) or 10 d (prostate), dormant tumor cells (breast d8; prostate d10) were observed. Over time (breast d18; prostate d22), these dormant tumor cells resumed growth, establishing “recurrent” colonies. **B.** SUM159 breast tumor cells (Parental, left panel; 4X) were incubated with Docetaxel (100 nM; 100 fold IC_50_) for 2 d, after which chemotherapy was removed and fresh culture medium added. Residual tumor cells were imaged on d8 after treatment (Residual tumor cells, middle panel; 4X). Colonies evolving from residual tumor cells were imaged on d18 (“Recurrent” colonies, right panel; 4X). Similar results were obtained using SUM159 cells incubated with Doxorubicin (Dox) for 2 d (1 µg/ml; 100 fold IC_50_; data not shown). **C.** DU145 prostate cancer cells (Parental, left panel; 4X) were incubated with Docetaxel (10 nM) for 4 d, after which chemotherapy was removed and fresh culture medium added. Residual tumor cells were imaged on d10 after treatment (Residual tumor cells, middle panel; 10X). Colonies were imaged on d22 (“Recurrent” colonies, right panel; 4X). **D.** SUM159 were incubated with Doxorubicin or Docetaxel as in “B”. Recurrent colonies were counted using crystal violet on d18. Likewise, DU145 cells were incubated with Docetaxel as in C. Recurrent colonies were counted using crystal violet on d22. Results are representative of at least three independent trials.

Several studies indicate that drug-resistant, slow-cycling tumor cells are represented at low frequency in human tumors, and are therapy resistant [5], [6]. The contribution of these cells to tumor recurrence following chemotherapy treatment is not known. We investigated the hypothesis that short-term exposure of tumor cells to chemotherapy enriches for a slow-cycling, chemo-resistant tumor cell sub-population that can, over time, resume growth, thus resembling tumor recurrence. To test this hypothesis, we exposed human breast (SUM159) and prostate (DU145) tumor cells to acute chemotherapy treatment (Fig. 1A). SUM159 breast tumor cells were exposed to Docetaxel (100 nM; 100-fold IC_50_) or Doxorubicin (1 µg/mL; 100-fold IC_50_). DU145 prostate tumor cells were exposed to Docetaxel (10 nM; 6-fold IC_50_). Chemotherapy was removed on d2 for SUM159 cells and on d4 for DU145 cells, and fresh culture medium was added. After 8 days (SUM159) or 10 days (DU145), the majority of tumor cells were dead. However, we noted that a small number of residual tumor cells remained (Fig. 1B and 1C). These residual tumor cells appeared to be non-proliferative, as indicated by the fact that their numbers did not increase for several days (data not shown). Approximately 10 d after chemotherapy removal, these residual tumor cells resumed proliferation (Fig. 3C) and eventually formed colonies, resembling a tumor recurrence (Fig. 1B–1D).

**Figure 2:**
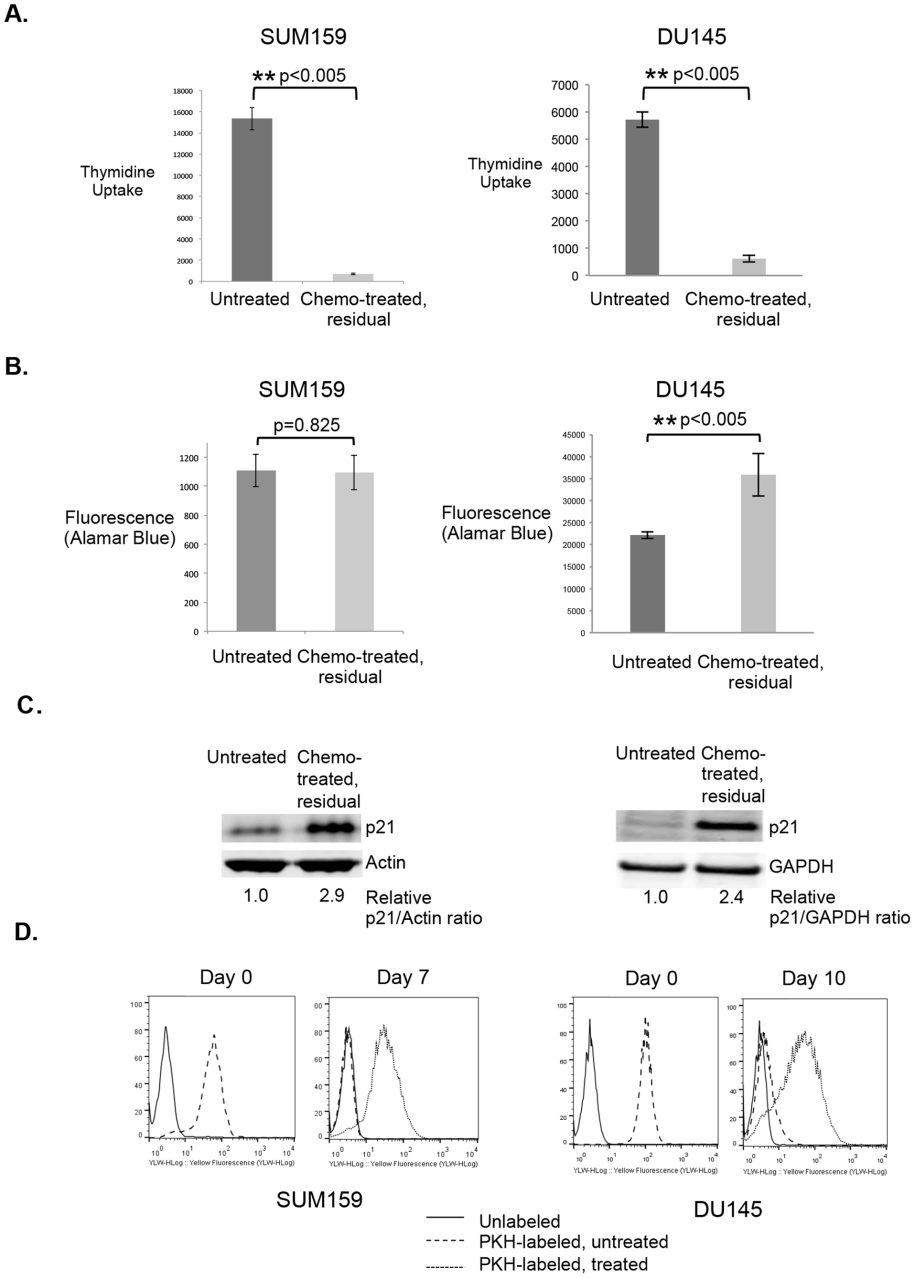
Chemotherapy enriches for dormant tumor cells. **A and B.** SUM159 breast and DU145 prostate cancer cells were exposed to acute Doxorubicin or Docetaxel treatment, respectively (as described in Fig. 1). Residual tumor cells surviving short-term chemotherapy treatment were harvested on d8 (breast) or d10 (prostate), and seeded at 2000 cells/well in triplicate wells of a 96 well plate. Proliferation was determined by thymidine incorporation (+/−SD). Cell viability was assessed by alamar blue (fluorescence +/− SD) (**B**). Statistical significance for (A) and (B) was determined using a two-tailed student’s t-test, with p<0.05 being considered significant. p≤0.05 (*); p≤0.005 (**). **C.** Total cellular protein was extracted from parental and residual, chemo-resistant tumor cells, and equivalent amounts were immunoblotted with p21 antibody, followed by IrDye-conjugated secondary antibody. Protein loading was assessed using Actin or GAPDH antibodies. Protein bands were detected by infrared imaging. Protein bands were quantified using Image J software (NIH), and the relative ratio of p21 to loading control is shown for each lane. Similar results were obtained in 3 independent trials. **D.** SUM159 or DU145 tumor cells were stained with the label-retaining dye PKH26, and labeling efficiency was assessed by flow cytometry on Day 0. PKH26-labelled SUM159 cells were either left untreated (- - - -) or incubated for 2 d with Doxorubicin (1 µg/ml; ––). PKH26-labelled DU145 cells were either left untreated (- - - -) or incubated for 4 d with Docetaxel (10 nM; ––). The % label-retaining cells was determined on d7 (SUM159) or d10 (DU145) after treatment. Note that at the time of harvest, the majority of untreated cells (proliferative) had lost the dye, whereas slow-cycling dormant cells enriched by chemotherapy had retained the dye.

Tumor dormancy has been defined as a condition in which residual cancer cells stop dividing [11]. It is thought that these cells remain dormant for a prolonged period before receiving signals (intrinsic or extrinsic) that cause them to resume growth and establish recurrent tumors. Fitting this definition of dormancy, both breast tumor cells and prostate tumor cells surviving short term chemotherapy in our model represented a sub-population of cells that did not take up appreciable thymidine (Fig. 2A), but were metabolically active, as indicated using an alamar blue assay (Fig. 2B). Notably, chemo-residual DU145 prostate cancer cells exhibited increased alamar blue positivity compared to parental DU145 cells, suggesting that these enriched cells may have elevated metabolism. Chemo-residual tumor cells also expressed increased levels of p21 (Fig. 2C), a cell cycle arrest protein. Contrasting with parental tumor cells, chemotherapy-enriched tumor cells were slow-cycling, as indicated by their retention of the lipophilic dye PKH26 (Fig. 2D).

**Figure 3:**
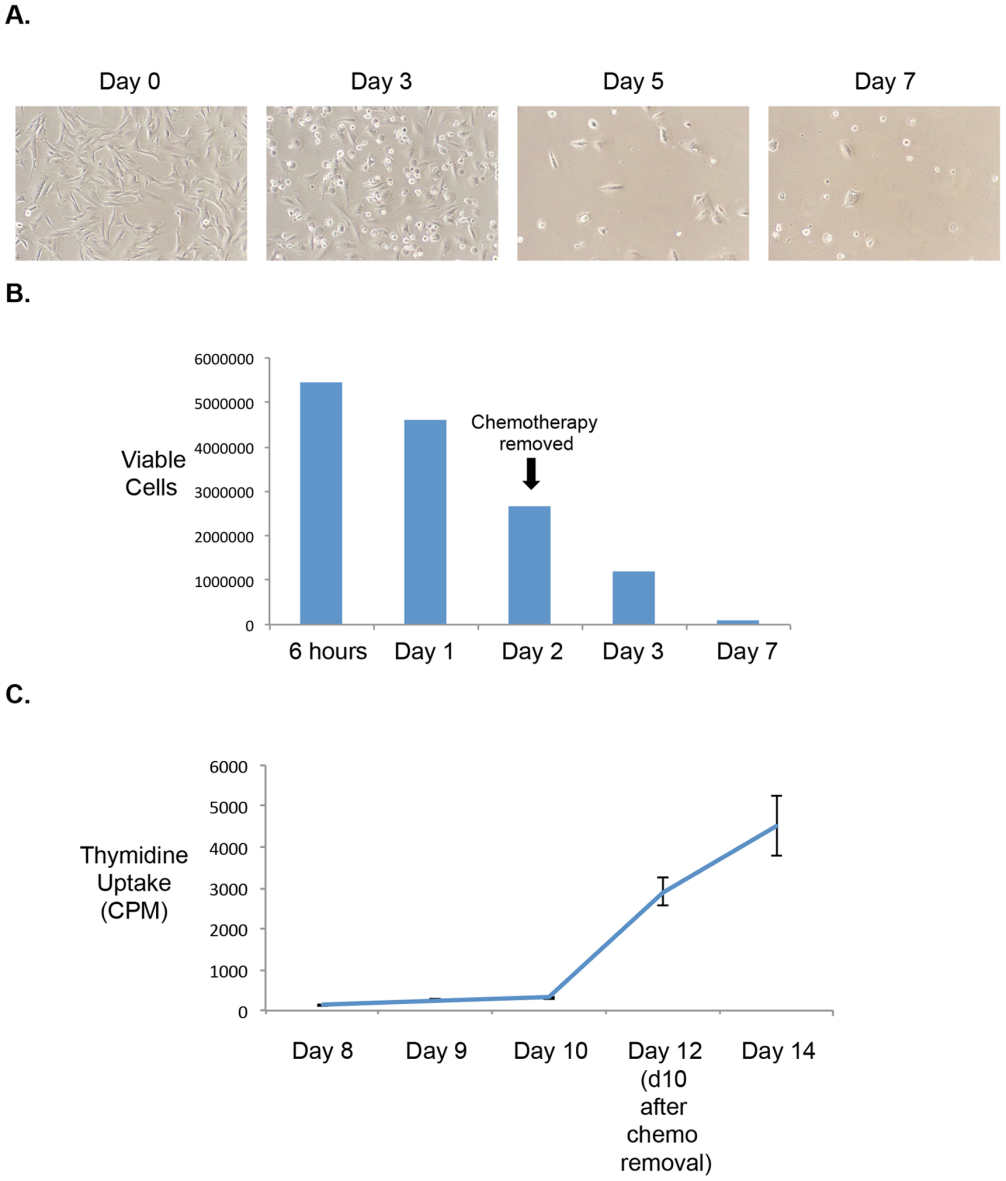
Kinetics of “recurrent” colony growth. SUM159 tumor cells were incubated with Doxorubicin (2d) as indicated in Fig. 1. **A and B.** Kinetics of cell die-off were assessed by imaging representative fields (**A**) as well as by counting viable cells using trypan blue (**B**) at the indicated times post-chemotherapy treatment. **C.** Proliferative status of residual tumor cells was measured over time by performing thymidine incorporation assays on cells (2000 cells/well) harvested at the indicated times post-chemotherapy treatment.

We next sought to determine the time after chemotherapy removal that dormant tumor cells resumed growth after chemotherapy removal. The number of viable breast tumor cells decreased for five days after chemotherapy removal, as demonstrated in Fig. 3A and B. However, residual tumor cells did not resume proliferation until approximately 10 days after chemotherapy removal, as assessed by thymidine uptake (Fig. 3C). Similar kinetics of growth were observed using the DU145/docetaxel prostate cancer model (data not shown).

**Figure 4:**
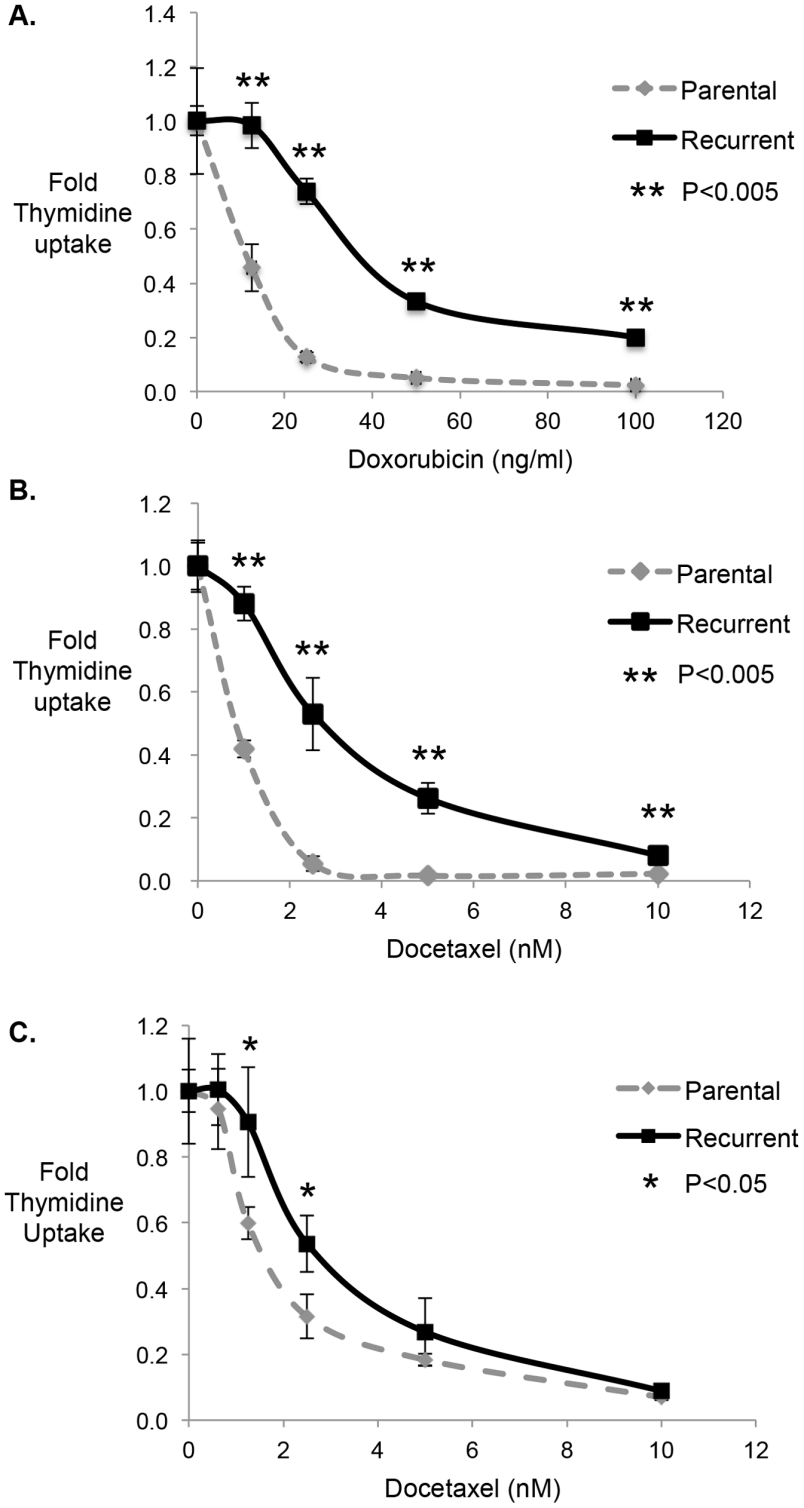
Tumor cells from recurrent colonies are more resistant to chemotherapy than parental tumor cells. **A and B.** SUM159 breast tumor cells were incubated with Doxorubicin (**A**) or Docetaxel (**B**) as in Fig. 1. Residual tumor cells were allowed to grow in the absence of chemotherapy, resulting in the evolution of “recurrent” colonies. Tumor cells from recurrent colonies, as well as parental tumor cells, were re-challenged with the indicated concentrations of Doxorubicin (**A**) or Docetaxel (**B**). Chemo-sensitivity was assessed by thymidine incorporation. Data for each point are expressed as fold change relative to cells cultured in media only. n = 4, error bars represent S.D., *p<0.05, **p<0.005. **C.** DU145 prostate tumor cells were incubated with Docetaxel as in Fig. 1. Residual tumor cells were allowed to grow in the absence of chemotherapy, resulting in the evolution of “recurrent” colonies. Tumor cells from recurrent colonies and parental tumor cells were re-challenged with the indicated concentrations of Docetaxel. Chemo-sensitivity was assessed by thymidine incorporation, as in A and B.

Recurrent tumors are frequently detected in cancer patients many years after initial chemotherapy treatment, and these tumors are chemo-refractory. Similar to recurrent tumors in patients, recurrent tumor cells evolving in our model from chemotherapy-enriched dormant cells exhibited increased chemotherapy resistance (Fig. 4). Increased therapy resistance was observed in both recurrent breast tumor cells (Fig. 4A and B) and in recurrent prostate tumor cells (Fig. 4C). Notably, resistant recurrent breast tumor colonies were observed independent of the class of chemotherapy treatment (taxane vs anthracycilne) (Fig. 4A and 4B).

## Discussion

Our results demonstrate that dormant, chemo-resistant tumor cells can be enriched from human breast and prostate tumor cell lines by short-term chemotherapy treatment. DNA-damaging (Doxorubicin) and microtubule-modifying (Docetaxel) chemotherapies, representing standard treatment regimens for breast and prostate cancer patients respectively, enriched for these dormant cells at clinically relevant doses [12], [13], indicating broad relevance to patient treatment (Fig. 1).

The current study focused on the ability of these dormant tumor cells to resume growth upon chemotherapy withdrawal, resembling the process of tumor recurrence. Notably, “recurrent” tumor cells evolving after chemotherapy withdrawal were more resistant to subsequent chemotherapy challenge than parental tumor cells. The therapy resistance of recurrent tumor cells in our model resembles therapy resistance of recurrent tumors in cancer patients [4].

The resistant phenotype of “recurrent” tumor cells evolving from our chemotherapy-enriched dormant cells contrasts with the reversibly-resistant phenotype of tumor cells subjected to long-term drug selection [6], [14]. To date, we have observed continued resistance of our “recurrent” breast tumor lines for 50 days after chemotherapy withdrawal (representing approximately 40 doubling times for these cells; data not shown). The irreversible resistance of these drug resistant tumor cells has important implications for patient treatment. Specifically, the existence of irreversible drug resistant phenotypes in the original tumor argues against models suggesting that recurrent tumors arising in patients after a gap in treatment (“drug holiday”) may benefit from retreatment with the same therapy [4]. Studies are ongoing to determine if “recurrent” tumor cells from our *in vitro* model remain chemo-refractory for months after therapy withdrawal.

We are currently defining resistance mechanisms (DNA repair, drug efflux) of recurrent tumor cells evolving from our short term chemotherapy enrichment model. Notably, recurrent colonies exhibiting increased chemotherapy resistance relative to parental tumor cells were obtained regardless of the chemotherapy class studied [DNA-damaging (Doxorubicin) or microtubule-modifying (Taxane)]. This finding raises the important possibility that chemo-resistant tumor cells may be cross-resistant to multiple chemotherapy classes, a topic of current investigation.

Our *in vitro* model of tumor dormancy/recurrence is important because it enriches for a dormant tumor cell population that is normally under-represented in the parental tumor cell line. Current studies in the lab are focused on identifying novel signaling pathways that drive tumor dormancy/recurrence using this short-term chemotherapy enrichment strategy. These studies have the potential to identify: 1) logical therapeutic targets in chemo-resistant, dormant tumor cell populations, and 2) biomarkers that predict recurrence-free survival.
